# An oncologist׳s friend: How *Xenopus* contributes to cancer research

**DOI:** 10.1016/j.ydbio.2015.02.003

**Published:** 2015-12-15

**Authors:** Laura J.A. Hardwick, Anna Philpott

**Affiliations:** Department of Oncology, University of Cambridge, Hutchison/MRC Research Centre, Cambridge Biomedical Campus, Cambridge CB2 0XZ, UK

**Keywords:** *Xenopus*, Cancer, Oncogenesis, Tumour, Model

## Abstract

One of the most striking features of the *Xenopus* system is the versatility in providing a unique range of both *in vitro* and *in vivo* models that are rapid, accessible and easily manipulated. Here we present an overview of the diverse contribution that *Xenopus* has made to advance our understanding of tumour biology and behaviour; a contribution that goes beyond the traditional view of *Xenopus* as a developmental model organism. From the utility of the egg and oocyte extract system to the use of whole embryos as developmental or induced tumour models, the *Xenopus* system has been fundamental to investigation of cell cycle mechanisms, cell metabolism, cell signalling and cell behaviour, and has allowed an increasing appreciation of the parallels between early development and the pathogenesis of tumour progression and metastasis. Although not the prototypical oncological model system, we propose that *Xenopus* is an adaptable and multifunctional tool in the oncologist׳s arsenal.

## Highlights

• *Xenopus* provides a unique *in vitro* and *in vivo* model for cancer research.
• Extract assays can model aspects of cell signalling, replication and metabolism.
• Many parallels exist between early development and tumour pathogenesis.
• Induced tumours in tadpoles can model aspects of human malignancies.
• *Xenopus* can contribute to improving cancer diagnostics and therapeutics.

## Introduction: the versatile *Xenopus* system applied to oncology

Since the 1950s, *Xenopus laevis* has become the most widely used amphibian research organism (Schmitt et al., 2014), with unique versatility in providing a range of both *in vitro* and *in vivo* models that are rapid, accessible and easily manipulated. The collective use of *Xenopus* oocytes, egg extracts, cell cultures and whole embryos in cancer research has made valuable contributions to understanding tumour biology, as well as improving therapeutic options in both diagnostics and chemotherapeutics.

## Modelling with *Xenopus* eggs and cleavage stage embryos

### Investigating cell cycle mechanisms

The events and regulatory mechanisms governing the cell cycle are essential for regulation of cellular proliferation and genomic stability, and several checkpoints exist to ensure successful completion of one stage before progression to the next (Harper and Brooks, 2005). Not only are pathways involved in cell cycle control frequently mutated in cancer, but deregulation of the cell cycle checkpoints promotes further acquisition of DNA mutations that can progress a cell down the route to metastasis (Laiho and Latonen, 2003).

*Xenopus* oocytes, eggs and early embryos have served as fundamental experimental systems in which to elucidate the mechanisms of the cell cycle and the coordination of the cell cycle and differentiation during development (e.g. Ali et al., 2011, Felix et al., 1990, Felix et al., 1989, Minshull et al., 1990; for a review, see Philpott and Yew, 2008). Large volumes of extracts can be prepared from eggs and oocytes by centrifugation, and these contain vast arrays of proteins that enable reconstitution of cell cycle events, nuclear transportation, microtubule polymerisation and apoptosis (e.g. Blow and Laskey, 1986, Laskey et al., 1978, Leno et al., 1992, Nutt, 2012, Zylkiewicz and Stukenberg, 2014, Deming and Kornbluth, 2006). Moreover, the cell-free nature of the extract system means the molecular machinery involved in these processes can be probed by manipulation of extracts with immune-depletion or neutralisation of an endogenous protein of interest, followed by subsequent rescue by addition of recombinant protein (e.g. Srinivasan and Gautier, 2011, Yew and Kirschner, 1997). Similar approaches allow the identification of endogenous targets of drug compounds that influence cell cycle kinetics (Rosania et al., 1999). Extracts can also be prepared to represent different phases of the cell cycle, and these, for example, can be used to study changes in protein stability during the cell cycle (McDowell et al., 2014, Vosper et al., 2009).

Given this impressive versatility, it is not surprising that the *Xenopus* egg extract system can also be applied to study the molecular derangements of cell cycle events that promote or accompany neoplastic transformation. In particular, biochemical investigation of the control of DNA replication, of DNA repair and of checkpoint control has been extensive. Plasmid replication can be studied in “nucleus-free” systems, but classic DNA replication experiments involve the addition of demembranated sperm chromatin to unfertilised egg extracts, which results in the formation of nuclei that undergo semi-conservative, cell-cycle regulated DNA replication (Leno and Laskey, 1991, Srinivasan and Gautier, 2011). Using this system, work has characterised the biochemical aspects of control of the replication process (Blow, 2001, Dikovskaya et al., 2012) and DNA replication fork stability (Hashimoto and Costanzo, 2011). Addition of linear DNA fragments to *Xenopus* egg extracts mimics the effects of double strand breaks in genomic DNA, enabling study of DNA damage checkpoints in a cell-free model (Willis et al., 2012, You et al., 2007). Detailed methods are also described for studying mitotic spindle assembly and checkpoints (Desai et al., 1999), and many mitotic spindle factors that are altered in cancer are conserved in *Xenopus* (Cross and Powers, 2009, Joukov et al., 2006).

However, *Xenopus* is not limited to *in vitro* investigation of cell cycle function; the developing *Xenopus* embryo also presents an interesting *in vivo* system to study regulation of proliferation (Woodland, 1974) particularly in view of the changes in the cell cycle regulation during early development (Saka and Smith, 2001). The first 12 embryonic cell cycles occur rapidly and synchronously, driven by stockpiles of maternal proteins, and alternating between DNA replication in S phase and cell division in M phase (Philpott and Yew, 2008). The mid-blastula transition (MBT) marks the onset of zygotic transcription, after which cell cycles become asynchronous and gradually lengthen with incorporation of G1 and G2 phases (Newport and Kirschner, 1982a, Newport and Kirschner, 1982b). Furthermore, the cell cycle is intimately linked with the process of differentiation during development (for example Carruthers et al., 2003, Vernon et al., 2006) and for review see (Hardwick and Philpott, 2014). As well as providing a platform for studying *in vivo* mechanisms of cell cycle control, the ease of experimental manipulation lends itself to investigate the mechanistic links between proliferation and differentiation, links that are often perturbed in cancer (Vernon et al., 2003, Richard-Parpaillon et al., 2004, Ali et al., 2011, Hindley et al., 2012).

### Using *Xenopus* oocytes to model cancer cell signalling

Distinct from studying somatic cell division, the *Xenopus* oocyte has also proven to be a highly versatile and powerful tool for investigating signalling cascades and their effects on cell growth. The large size of the oocyte enables microinjection of substances without disturbance of the normal physiology (Woodland, 1974) and components of signalling paths can be studied in isolation from the complex milieu of growth signals that complicate the endogenous tumour environment (Cailliau et al., 2005, Browaeys-Poly et al., 2009). For example, tumour-secreted fibroblast growth factor 1 (FGF1) and tumour-expression of its receptor FGFR1 are involved in the proliferation of oestrogen-negative human breast cancer cells (Cailliau et al., 2005). *Xenopus* oocytes lack endogenous FGFRs, enabling the study of FGFR1 receptors derived from malignant breast cancer cell lines following oocyte over-expression. Stimulation of the exogenous receptors by *in vitro* applied FGF ligands triggers oocyte maturation and passage through the G2/M transition that serves as a read-out of FGF signal transduction (Cailliau et al., 2005). This model has been used to characterise the downstream components responsible for this transition into M phase (Browaeys-Poly et al., 2009), and also to identify potential pharmacological inhibitors of this pathway (Cailliau et al., 2005).

### *Xenopus* oocytes and cancer metabolism

The *Xenopus* oocyte has also found favour in the studies of cancer metabolism. In this respect, the oocyte has the advantage of being large enough to enable biochemical measurements in a single cell, and allow microinjection of compounds that would otherwise require the use of harsh permeabilisation techniques in heterogenous populations of cells (Ureta et al., 2001). Indeed, the *Xenopus* oocyte has been described as a “living test-tube” in which to study metabolic regulation (Ureta et al., 2001). It is perhaps not surprising therefore, that the oocyte has also formed an *in vitro* model for the study of deranged metabolism in cancer cells. A detailed discussion is beyond the scope of this review, but interestingly, the metabolic phenotypes and intermediary pathways seen in cancer cells are similar to those found in the *Xenopus* oocyte, enabling the extract system to model the relationship between cancer metabolism and cell death (Deming and Kornbluth, 2006, Dworkin and Dworkin-Rastl, 1989) and for review see (Nutt, 2012). Furthermore, over-expression of tumour-associated signalling proteins and nutrient transporters in *Xenopus* oocytes can serve as a model to study alterations in glucose handling by tumour cells, (for example Pakladok et al., 2012). Overall, these *Xenopus* assays have already contributed to cancer metabolomics but also provide viable models for future investigations into therapeutic targeting of abnormal cancer metabolism.

## *Xenopus* embryogenesis: a developmental model for cancer studies

### Parallels between early development and tumourigenesis

From as early as the 1890s, parallels were drawn between the development of the early embryo and the pathological development of neoplasia. Over a century of research has built on these foundations and a body of literature now documents the similarities in terms of epigenetic signatures, transcriptomes, proteomes, metabolism and cell behaviour (Ma et al., 2010, Wang, 2009, Wu et al., 2007, Xie and Abbruzzese, 2003). Signalling pathways that are critical during embryonic development (such as Sonic Hedgehog, Wnt, Notch and Bone Morphogenetic Proteins) are also instrumental in tumour progression to a metastatic phenotype (Bailey et al., 2007). Similarly, high rates of cell proliferation are seen in both embryonic and tumour cells, in part due to the activity of pro-proliferative signalling pathways and transcription factors (Ma et al., 2010). Therefore cancer can perhaps be viewed as an inappropriate re-activation or alteration of normal embryonic growth pathways (Pennisi, 1998), or perhaps a disorder of cellular differentiation, where cells instead remain locked in the proliferative mode prior to cell fate determination, as seen in some induced tumour models (Wallingford et al., 1997). In this respect, characterisation of normal developmental pathways can assist in understanding the derangements that occur during oncogenesis, and also aid in the identification of potential novel diagnostic or therapeutic targets. Acknowledged benefits of the *Xenopus* system include the accessibility of the early developmental stages and the ease of targeted expression due to accurate fate maps. These enable *Xenopus* to form an instrumental model for developmental biologists and oncologists alike (Wallingford, 1999).

### Characterising oncogenes: induced tumours in developing *Xenopus* embryos

In the late 1990s, three independent groups reported induced tumour phenotypes in developing *Xenopus* embryos. This was achieved either through over-expression of oncogenes such as Gli1 (Dahmane et al., 1997) or Xrel3 (Yang et al., 1998), or through inhibition of tumour suppressor protein p53 (Wallingford et al., 1997). Histologically these induced tumours contain poorly differentiated cells with abnormal nuclear morphologies (Wallingford, 1999), and in the case of Gli1-induced epidermal growths, the molecular phenotype suggests that these tadpole tumours may be equivalent of human basal cell carcinomas (Dahmane et al., 1997). Subsequent work has referred to these lesions as induced tumour-like structures (ITLS), characterised by disorganised undifferentiated cells with increased proliferation and abnormal nuclear size, invasive behaviour and the ability to stimulate angiogenesis (Chernet and Levin, 2014). Thus, from this early work, *Xenopus* has gained increasing credibility as a valid *in vivo* model for various forms of human malignancy, in addition to the potential for functional screening for therapeutic agents that may diminish the induced tumour phenotype (Wallingford, 1999).

Interestingly, the early work that uncovered a tumourigenic phenotype from inhibition of p53 in *Xenopus* embryos, was originally undertaken to characterise the role of p53 during early embryogenesis. *Xenopus* was used as a rapid developmental model to minimise the effects of genetic instability resulting from p53 knockdown (Wallingford et al., 1997). Tumour cells divide normally, but fail to undergo the normal differentiation process (Wallingford et al., 1997), and this may therefore support the view that cancer is sometimes a failure of differentiation rather than abnormal proliferation *per se*. Similarly, the dose-dependent tumourigenic phenotype from over-expression of Xrel3 was realised following identification of this novel c-rel homologue during early development; the authors suggest that the endogenous Xrel3 function may be in the cellular proliferation *versus* differentiation decision (Yang et al., 1998).

These examples highlight the importance of examining the parallels between development and tumourigenesis, not only to uncover cellular functions for known oncogenes, but also to assist identification of potential oncogenic activity in genes with a known developmental function. In this way, viral and cellular proto-oncogenes have been found to have endogenous developmental roles, for example as p21^ras^ (Whitman and Melton, 1992). Conversely, human homologues of developmental regulators have been identified as having key oncogenic or tumour suppressor roles. For example, the presence of anterior gradient 2 (AGR2), a human homologue of the *Xenopus* cement-gland specific gene, correlates with and can even confer metastatic phenotype in human breast carcinomas (Liu et al., 2005), whereas VentX, the human homologue of *Xenopus* homeobox transcription factor Xom, induces senescence in osteosarcoma and lymphoblastic leukaemia cell lines (Wu et al., 2011).

Developing *Xenopus* embryos can also be used to probe the molecular mechanisms of oncogenes in signalling pathways. For example, HGF/SF (hepatocyte growth factor scatter factor) is a ligand for activation of the Met tyrosine kinase receptor, and aberrant activity of this pathway contributes to tumour cell invasion and metastasis in a number of human cancers (Ishimura et al., 2006). Over-expression of oncogenic mutant forms of Met receptor in *Xenopus* embryos leads to the formation of ectopic morphogenic structures, thereby providing a rapid *in vivo* assay of tumorigenesis. This model has enabled identification of downstream components that contribute to the oncogenic deregulation of this pathway (Ishimura et al., 2006).

In recent years, several powerful genome editing techniques have become available for use in *Xenopus*, opening the way to advanced genetic modifications that are already established in murine models; these include the use of zinc-finger nucleases (ZFNs), transcription activator-like effector nucleases (TALENs) and CRISPR/Cas system, as reviewed in (Schmitt et al., 2014). Combined with the expanding number of transgenic frog lines, it will surely not be long before transgenic models are generated to model various aspects of specific mammalian cancers. Such a technique has recently been employed to produce a transgenic *Xenopus* model to study lymphangiogenesis (Ny et al., 2005, Ny et al., 2013). With an increasingly appreciation of the underlying genetic derangements in specific types of human cancers, this will be an interesting avenue to pursue with *Xenopus* transgenics.

### Wnt signalling in development and cancer

The Wnt signalling pathway has vital roles at different stages in embryonic development, regulating processes including cell proliferation and cell fate specification, cell migration and polarity, and body axis formation (Hikasa and Sokol, 2013). Yet aberrant activity of Wnt ligands and their downstream effectors are also important for tumour initiation, growth, progression and metastasis, with acknowledged roles in breast, prostate, gastro-intestinal, liver and lung cancers, in addition to melanoma and bone metastasis (Xi and Chen, 2014, Tumova et al., 2014).

**Fig. 1:**
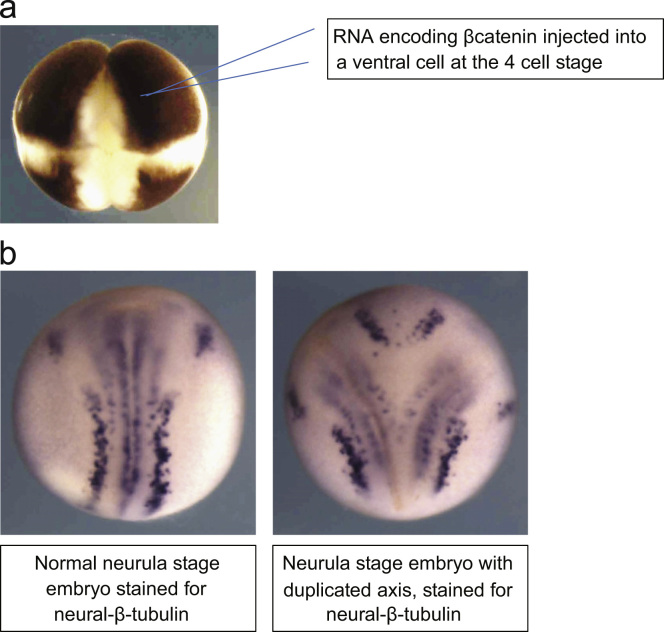
(A) A secondary axis can be induced in developing *Xenopus* embryos by injection of RNA encoding β-catenin into a ventral cell of 4-cell stage embryos. Ventral cells are usually distinguished by their larger size and darker pigment compared to dorsal cells. For detailed methods see (Kuhl and Pandur, 2008a). (B) The duplicated axis is visible in neurula stage embryos within 2 days of injection. Embryos in these images have undergone *in situ* hybridisation for neural-β-tubulin to illustrate the bilateral stripes of primary neurons and trigeminal ganglia. Embryos can be exposed to a range of compounds during development to assay for ability of the compound to inhibit axis duplication. Alternatively, RNA encoding proteins of interest can be injected into the ventral cells to assay for ability of the protein to induce a secondary axis.

Different intracellular signalling cascades can be activated downstream of the active Wnt-Frizzled receptor, in part due to the diversity of Wnt ligand families that primarily activate one or more pathways and have conflicting effects on cell behaviour (Gradl et al., 1999). Canonical Wnt signalling refers to activation of the Wnt/β-catenin pathway by Wnt-1 family ligands. Non-canonical Wnt pathways include activation of the Wnt/Ca^2+^ pathway and the Wnt/planar cell polarity (PCP) pathway, usually by Wnt-5A family members (Kuhl, 2002). These signalling cascades are highly conserved in evolution, and *Xenopus* has provided rapid assay methods to investigate various aspects of pathway regulation. The most well-established assay for canonical Wnt signalling is the *Xenopus* axis duplication assay (Fig. 1), but *Xenopus* oocytes can also be used as a secretory system to release human Wnt ligands for a variety of paracrine assays (Cha and Heasman, 2010), and CaMKII activity in *Xenopus* embryos can provide a measure of non-canonical Wnt signalling (Kuhl and Pandur, 2008b).

Given the instrumental role that aberrant Wnt signalling can have in cancer progression, inhibition of this pathway has proved to be an attractive chemotherapeutic target, and the *Xenopus* axis duplication assay is a highly efficient screen for potential candidate inhibitors (see below and Kuhl and Pandur, 2008a). Endogenously, β-catenin is stabilised on the prospective dorsal side of the embryo during the first cell cycle, due to the cortical rotation that follows sperm entry. This leads to activation of transforming growth factor-β (TGFβ) family members *siamois* and *Xenopus nodal-related 3* (*Xnr-3*), that promote formation of dorsal structures. Injection of mRNA encoding Wnt ligand or β-catenin into the marginal zone of a ventral blastomere of a 4-cell stage *Xenopus* embryo results in duplication of the body axis, with results visible from neurula stages, just 36 h after injection (Kuhl and Pandur, 2008a).

### Epithelial to mesenchymal transitions in development and cancer

One of the most striking examples of the similarity between embryonic development and malignant behaviour is presented by the morphological changes that accompany epithelial to mesenchymal transitions (EMT) in development and cancer. These EMT and mesenchymal to epithelial (MET) events are fundamental changes of cell shape, polarity and migration that are central but transient events during embryogenesis, and are responsible for processes such as blastula formation, gastrulation, neural crest formation, somitogenesis and cardiac morphogenesis; for detailed reviews see (Acloque et al., 2009, Acloque et al., 2008, Kalluri and Weinberg, 2009, Nakaya and Sheng, 2013, Theveneau and Mayor, 2012, Thiery et al., 2009). These transitions may be complete or partial during embryogenesis, but nevertheless they provide essential insights into behaviours that can be inappropriately reactivated during tumour progression, while EMT is a key event in malignant invasion (Vernon and la Bonne, 2004). Indeed, genes that have pivotal roles in EMT events during embryogenesis are often mutated or aberrantly expressed in cancer cells (Lander et al., 2013, Mani et al., 2007, Nakaya and Sheng, 2013, Taube et al., 2010, Vernon and la Bonne, 2004, Yang et al., 2004); this underscores the importance of understanding the developmental regulation of this process.

In this way, study of embryonic neural crest cells is often used as an *in vivo* and *in vitro* model of EMT, with neural crest cells displaying the activity of signalling paths, transcription factors, cell-adhesion changes and matrix remodelling that are similarly responsible for invasion and metastasis of epithelial tumours, comprehensively reviewed in (Theveneau and Mayor, 2012). This developmental approach has highlighted the importance of the bHLH protein Twist, for both embryonic and cancer-associated EMT (Vernon and la Bonne, 2004, Yang et al., 2004), with *Xenopus* neural crest formation serving as a model to investigate the role and regulation of Twist (Lander et al., 2013). Twist is similarly considered a master regulator of EMT in malignant breast cancer, highly expressed in tumour cells yet rarely expressed in normal adult cells, therefore generating interest as a potential therapeutic target (Glackin, 2014). Additionally, the study of epithelial cell behaviour can also be conducted using the established *Xenopus* A6 cell line. These cells are derived from normal *Xenopus* kidney tissue, and behave *in vitro* as typical polarised epithelial monolayers, and therefore are suitable for investigating epithelial cell migration and morphogenesis (Mimori-Kiyosue et al., 2007).

Taken together, these examples highlight the complementary fields of developmental biology and oncology, and how advances in one area can prove directly relevant to understanding the molecular mechanisms in the other. Signalling pathways and morphogenic events in development are highly conserved between species; *Xenopus* is therefore a highly pertinent model for elucidating these critical components.

## Spontaneous tumours and tumour resistance in *Xenopus*

Spontaneous tumours in amphibians are rare (Ruben et al., 2007), but even this phenomenon can nevertheless make a valuable contribution to mammalian cancer biology. The multi-step process through which a cell progresses in order to gain a fully malignant phenotype has been well characterised in mammals, both at cellular and genetic levels (Hanahan and Weinberg, 2000, Hanahan and Weinberg, 2011). What are less well characterised are the complex and multiple interactions that exist between tumour cells and the host immune system. In one respect, “cancer immune-surveillance” describes the detection and destruction of tumour cells by the host immune system. Conversely, the immune system can exert a selection pressure on the heterogenous tumour cell population, leading to the persistence and growth of resistant or less immunogenic cells in the process of “immune-editing” (Bui and Schreiber, 2007). *Xenopus* has played a key role in both *in vitro* and *in vivo* studies of anti-tumour immune responses, providing evidence for both of these mechanisms (Goyos and Robert, 2009).

### Spontaneous tumours

Despite the relatively lower incidence of spontaneous tumours in *Xenopus* compared to mammalian models, several neoplastic conditions have been described, including hepatomas (Robert, 2010), ovarian tumours (Goyos and Robert, 2009), and thyroid-containing teratomas (Cheong et al., 2000). The definition of neoplasia in *Xenopus* has come under some scrutiny, as the original report of a virally-induced and highly malignant lymphosarcoma (Balls, 1965) was subsequently shown to be a transmissible (but not transplantable) infectious granuloma (Asfari and Thiebaud, 1988), caused by myocobacterium marinum (Asfari, 1988). However, the most experimentally useful tumours have proven to be the thymic lymphomas, first recorded in the early 1990s and subsequently described in 4 genetically different adult *Xenopus* frogs at the Basel Institute of Immunology (du Pasquier and Robert, 1992, Robert et al., 1994), and one at the Tulane University *Xenopus* colony (Earley et al., 1995). The tumours fulfil the criteria for spontaneous neoplasia, propagating in isogenic animals, being rejected in allogenic animals and enabling the derivation of stable lymphoid cell lines that can be cloned from single cells and cultured indefinitely (du Pasquier and Robert, 1992). Characteristics of the 5 lymphoid cell lines (B3B7, 15/40, 15/0, ff-2 and ff2.64) are comprehensively reviewed elsewhere (Robert and Cohen, 1998), and while all are mixed T/B cell phenotypes, they appear to arise from independent oncogenic events (Robert et al., 1994). These cell lines also demonstrate aneuploidy with marked genetic instability in addition to up-regulation of c-myc (Goyos and Robert, 2009), thus illustrating their relevance as *in vitro* and *in vivo* models of mammalian cancer biology.

### Tumour resistance in amphibians

The relative resistance of *Xenopus* (and other amphibians) to spontaneous and transplanted tumours has provided an intriguing model to study the mechanisms of tumour immunity. A range of developmental and physiological features in amphibians may contribute to their relative tumour resistance, as reviewed in (Ruben et al., 2007), and metamorphosis provides a particularly interesting evolutionary perspective with respect to differences between the more primitive larval immune system and that found in the adult (Robert and Cohen, 1998). The stable lymphoid cell lines derived from the different thymic tumours have been used in transplantation studies to determine components of the laval or adult immune system that are required to afford tumour immunity (du Pasquier and Robert, 1992, Robert et al., 1997). Together, these studies have demonstrated a conserved and critical function of T cells in tumour immunity, directed against tumour-specific antigens (Robert et al., 1997). Furthermore, recent work has identified molecular mechanisms involving expression of class Ib MHC molecules that may be responsible for immune evasion by the 15/0 tumour cells (Haynes-Gilmore et al., 2014). In combination, these studies support both the immune-surveillance and immune-editing hypotheses, and it will now be interesting to translate these findings to comparable mammalian tumour models.

## *Xenopus* contributions to translational medicine: insights that may improve clinical approaches to cancer

From the discussion above, the *Xenopus* system has clear relevance to our understanding of cancer aetiology, biology and physiology, and from these studies, future therapeutic targets may be identified. This final section focuses more specifically on the application of the *Xenopus* system to clinical practice, largely in the form of developing chemotherapeutic agents, but we finish with a novel and exciting avenue of *Xenopus* research into epigenetic reprogramming of cancer cells.

### Chemotherapeutic drug discovery

The contributions of the *Xenopus* system to drug discovery and development again emphasises the versatility of this system, with oocytes, isolated embryonic cells and whole embryo models available to researchers. *Xenopus* oocytes have served as a fundamental tool in pharmaceutical research, from preliminary drug candidate screening (Kvist et al., 2011, Landais et al., 2009) through to characterisation of drug pharmacodynamics (Wei et al., 2013) and drug pharmacokinetics and tumour targeting (Anderson et al., 2010). This model has been used extensively to study the electrophysiology of exogenous ion channels, and the activity of compounds to modulate channel function in the search for new chemotherapeutics, for example (Kvist et al., 2011). Injection of *in vitro* transcribed cRNAs into oocytes can produce functional channel expression within 2 days, and the large size of the oocyte is readily amendable to patch clamp experiments or 2 electrode voltage clamping (Liu et al., 2002, Pakladok et al., 2014). This approach can also aid advances in diagnostic imaging, for example in characterising the kinetics of radiotracers used in PET (positron emission tomography) scans for visualising prostate cancer (Okudaira et al., 2013).

Secondly, isolated embryonic cells provide a rapid and efficient model for assaying the anti-proliferative effects of potential chemotherapeutics (Miyata et al., 2004), and the utility of the whole embryo axis duplication assay was mentioned earlier as a vital screen for compounds affecting the canonical Wnt signalling path (Kuhl and Pandur, 2008a). These studies have led to identification of candidate drugs that may assist in the future treatment of several human cancers, particularly colon cancer (Tumova et al., 2014, Waaler et al., 2012, Waaler et al., 2011) and glioblastoma (de Robertis et al., 2013). Additionally, older tadpole stage embryos can also provide an assay method to easily assess the effects of compounds on organ development; progressive transparency acquired during tadpole stages enables direct visualisation of a range of organs and tissues. This has been utilised to identify chemicals that suppress pigment cell development with potential use in treatment of melanoma, and compounds with anti-angiogenic or anti-lymphangiogenic activity that may have relevance to inhibit tumour pathogenesis (Schmitt et al., 2014).

### *Xenopus* extracts and epigenetic reprogramming

Another interesting recent field of study has involved the possibility of reprogramming cancer cells in order to reverse the epigenetic changes that have resulted in gene activation or silencing at certain critical gene promoters. From the pioneering nuclear transfer experiments of John Gurdon and colleagues (Gurdon et al., 1958), the field of cellular reprogramming has advanced, with hopes of application to disease modelling and ethically acceptable forms of regenerative medicine. Indeed, several techniques are now recognised for directly or indirectly converting one somatic cell type to another, but a caveat remains regarding aberrant reprogramming that can be tumorigenic (Goding et al., 2014). Significant investment is being made to define cocktails of mammalian transcription factors that can direct lineage conversion on over-expression (Lujan and Wernig, 2012, Ali et al., 2014), but *Xenopus* also presents experimental systems for reprogramming by nuclear transfer (Halley-Stott et al., 2013).

In addition to the established genetic mutations that have been characterised in various human cancers, abnormal epigenetic alterations have been ascribed roles in the pathogenesis of several different human malignancies (Sadikovic et al., 2008). Epigenetics refers to stable and heritable patterns of gene expression that contribute to cellular phenotype, caused by mechanisms other than changes in primary DNA nucleotide sequences (Halley-Stott and Gurdon, 2013). However, epigenetic chromatin modifications are reversible and therefore attractive targets to counteract malignancy (Allegrucci et al., 2011). Extracts prepared from ovarian prophase axolotl oocytes have previously been shown to remodel somatic mammalian cell chromatin (Bian et al., 2009), and this work has since been extended to demonstrate reprogramming of breast cancer cell lines by axolotl and *Xenopus* oocyte extracts. Mechanistically, reactivation of silenced tumour suppressor genes is achieved through promoter demethylation and histone remodelling, and phenotypically this is associated with long term suppression of breast cancer cell tumourigenicity (Allegrucci et al., 2011). Furthermore, this phenomenon has similarly been demonstrated using bovine oocyte extracts to reprogram human lung cancer cells, reactivating silenced tumour suppressor genes without up-regulation of pluripotency-associated genes (Wang et al., 2013a, Wang et al., 2013b)

The application of this technology to cancer studies will be more as a method to study the epigenetic contribution to tumorigenesis, rather than a treatment *per se*. But future work may uncover mechanisms that can be therapeutically targeted to achieve a reversal of the epigenetic alteration, thus ameliorating the malignant phenotype.

## Concluding remarks: *Xenopus* as a complimentary system to mouse and human cancer models

The preceding discussion has revealed the large contribution that *Xenopus* has, and continues to make to mammalian oncology. From an understanding of the basics of cell division and differentiation, through oncogene function and cancer aetiology, to characterisation of the molecular pathogenesis and metabolic derangements of cancer, these insights from *Xenopus* may ultimately translate into therapeutic benefits in the form of new prospective diagnostic tests or chemotherapeutics. Although not the prototypical oncological model system, we propose that *Xenopus* is an adaptable and multifunctional tool in the oncologist׳s arsenal; a tool that compliments the more extensively used rodent models in cancer research.

Traditional approaches in rodent models have involved direct genetic manipulations, often resulting in the generation of mice harbouring oncogenic mutations or missing tumour suppressor genes or both (for instance Berry et al., 2012, Hingorani et al., 2005 but the list is endless). Alternatively, and sometimes complimentarily, tumours have been generated by chemical induction or through xenografted tumourigenic tissues, often from patient cancers (for review, see Cekanova and Rathore, 2014). These approaches have contributed to both the study of oncogenic mechanisms and also to provision of pre-clinical data prior to human trials.

Some advantages of these mammalian systems are obvious in terms of recapitulating the accumulation of multiple genetic mutations, complex tumour and stroma microenvironments, and immune regulation that are seen in human patients. However, mammalian models are not without their limitations in mimicking human disease, and indeed, the complexity of rodent models can itself hinder phenotypic analysis. For instance, cyclin-dependent kinases (cdks) play a central role in cell cycle progression, yet knock-out mouse models of even key cdks such as cdk2 and cdk4 result in a remarkably mild phenotype (Berthet et al., 2003, Ortega et al., 2003, Rane et al., 1999, Tsutsui et al., 1999). These highly unexpected findings have been shown to result from redundancy and/or facultative compensation between cdks (Santamaria and Ortega, 2006). Reduced complexity is one reason why *Xenopus* can triumph over rodents in such studies; for example, at early developmental stages studied, *Xenopus* has a single cdk inhibitor, p27Xic1, which has characteristics of all 3 mammalian Cip/Kip family cdkis (Vernon, 2003, Vernon and Philpott, 2003).

In cell division, differentiation and cancer, as in other areas, it is remarkable how almost all of what we see in *Xenopus* is recapitulated in mammalian cells and indeed *in vivo* in mice. This, once again, underscores the high degree of conservation of fundamental mechanisms amongst vertebrates, and it is clear that experiments in *Xenopus* can usually be undertaken on vastly shorter time-scales and at considerably smaller expense than those using mouse models. These facts, coupled with the animal welfare and ethical advantages of using eggs and tadpoles to replace rodents, means that *Xenopus* remains a vital and if somewhat underused weapon to provide initial observations that can then be extended into mammalian models of cancer. If scientists using *Xenopus* and mammalian models work together with a fuller understanding of the merits and drawbacks of both systems, our combined efforts will allow the maximum progress in the fight against cancer.

## References

[bib1] Acloque H., Adams M.S., Fishwick K., Bronner-Fraser M., Nieto M.A. (2009). Epithelial–mesenchymal transitions: the importance of changing cell state in development and disease. J .Clin. Investig..

[bib2] Acloque H., Thiery J.P., Nieto M.A. (2008). EMBO Rep.. The physiology and pathology of the EMT. Meeting on the epithelial–mesenchymal transition.

[bib3] Ali F., Hindley C., McDowell G., Deibler R., Jones A., Kirschner M., Guillemot F., Philpott A. (2011). Cell cycle-regulated multi-site phosphorylation of Neurogenin 2 coordinates cell cycling with differentiation during neurogenesis. Development.

[bib4] Ali F.R., Cheng K., Kirwan P., Metcalfe S., Livesey F.J., Barker R.A., Philpott A. (2014). The phosphorylation status of Ascl1 is a key determinant of neuronal differentiation and maturation *in vivo* and *in vitro*. Development.

[bib5] Allegrucci C., Rushton M.D., Dixon J.E., Sottile V., Shah M., Kumari R., Watson S., Alberio R., Johnson A.D. (2011). Epigenetic reprogramming of breast cancer cells with oocyte extracts. Mol. Cancer.

[bib6] Anderson C.M., Jevons M., Thangaraju M., Edwards N., Conlon N.J., Woods S., Ganapathy V., Thwaites D.T. (2010). Transport of the photodynamic therapy agent 5-aminolevulinic acid by distinct H+-coupled nutrient carriers coexpressed in the small intestine. J. Pharmacol. Exp. Ther..

[bib7] Asfari M. (1988). Mycobacterium-induced infectious granuloma in *Xenopus*: histopathology and transmissibility. Cancer Res..

[bib8] Asfari M., Thiebaud C.H. (1988). Transplantation studies of a putative lymphosarcoma of *Xenopus*. Cancer Res..

[bib9] Bailey J.M., Singh P.K., Hollingsworth M.A. (2007). Cancer metastasis facilitated by developmental pathways: sonic hedgehog, Notch, and bone morphogenic proteins. J. Cell. Biochem.

[bib10] Balls M. (1965). Lymphosarcoma in the South African clawed toad, *Xenopus laevis*: a virus tumor. Ann. N. Y. Acad. Sci..

[bib11] Berry T., Luther W., Bhatnagar N., Jamin Y., Poon E., Sanda T., Pei D., Sharma B., Vetharoy W.R., Hallsworth A., Ahmad Z., Barker K., Moreau L., Webber H., Wang W., Liu Q., Perez-Atayde A., Rodig S., Cheung N.K., Raynaud F., Hallberg B., Robinson S.P., Gray N.S., Pearson A.D., Eccles S.A., Chesler L., George R.E. (2012). The ALK(F1174L) mutation potentiates the oncogenic activity of MYCN in neuroblastoma. Cancer Cell 22.

[bib12] Berthet C., Aleem E., Coppola V., Tessarollo L., Kaldis P. (2003). Cdk2 knockout mice are viable. Curr. Biol..

[bib13] Bian Y., Alberio R., Allegrucci C., Campbell K.H., Johnson A.D. (2009). Epigenetic marks in somatic chromatin are remodelled to resemble pluripotent nuclei by amphibian oocyte extracts. Epigenet. Off. J. DNA Methylation Soc..

[bib14] Blow J.J. (2001). Control of chromosomal DNA replication in the early *Xenopus* embryo. EMBO J..

[bib15] Blow J.J., Laskey R.A. (1986). Initiation of DNA replication in nuclei and purified DNA by a cell-free extract of *Xenopus* eggs. Cell.

[bib16] Browaeys-Poly E., Perdereau D., Lescuyer A., Burnol A.F., Cailliau K. (2009). Akt interaction with PLC(gamma) regulates the G(2)/M transition triggered by FGF receptors from MDA-MB-231 breast cancer cells. Anticancer Res..

[bib17] Bui J.D., Schreiber R.D. (2007). Cancer immunosurveillance, immunoediting and inflammation: independent or interdependent processes?. Curr. Opin. Immunol..

[bib18] Cailliau K., Perdereau D., Lescuyer A., Chen H., Garbay C., Vilain J.P., Burnol A.F., Browaeys-Poly E. (2005). FGF receptor phosphotyrosine 766 is a target for Grb14 to inhibit MDA-MB-231 human breast cancer cell signaling. Anticancer Res..

[bib19] Carruthers S., Mason J., Papalopulu N. (2003). Depletion of the cell-cycle inhibitor p27(Xic1) impairs neuronal differentiation and increases the number of ElrC(+) progenitor cells in *Xenopus tropicalis*. Mech. Dev..

[bib20] Cekanova M., Rathore K. (2014). Animal models and therapeutic molecular targets of cancer: utility and limitations. Drug Des. Dev. Ther..

[bib21] Cha S.W., Heasman J. (2010). Using oocytes for Wnt signaling assays: paracrine assays and Wnt-conditioned medium. Methods.

[bib22] Cheong S.W., Fukui A., Asashima M., Pfeiffer C.J. (2000). Spontaneous thyroid-containing teratoma associated with impaired development in the African clawed frog, *Xenopus laevis*. J. Comp. Pathol..

[bib23] Chernet B.T., Levin M. (2014). Transmembrane voltage potential of somatic cells controls oncogene-mediated tumorigenesis at long-range. Oncotarget.

[bib24] Cross M.K., Powers M.A. (2009). Learning about cancer from frogs: analysis of mitotic spindles in *Xenopus* egg extracts. Dis. Models Mech..

[bib25] Dahmane N., Lee J., Robins P., Heller P., Ruiz i Altaba A. (1997). Activation of the transcription factor Gli1 and the Sonic hedgehog signalling pathway in skin tumours. Nature.

[bib26] de Robertis A., Valensin S., Rossi M., Tunici P., Verani M., De Rosa A., Giordano C., Varrone M., Nencini A., Pratelli C., Benicchi T., Bakker A., Hill J., Sangthongpitag K., Pendharkar V., Liu B., Ng F.M., Then S.W., Jing Tai S., Cheong S.M., He X., Caricasole A., Salerno M. (2013). Identification and characterization of a small-molecule inhibitor of Wnt signaling in glioblastoma cells. Mol. Cancer Ther..

[bib27] Deming P., Kornbluth S. (2006). Study of apoptosis *in vitro* using the *Xenopus* egg extract reconstitution system. Methods Mol. Biol..

[bib28] Desai A., Murray A., Mitchison T.J., Walczak C.E. (1999). The use of *Xenopus* egg extracts to study mitotic spindle assembly and function *in vitro*. Methods Cell Biol..

[bib29] Dikovskaya D., Khoudoli G., Newton I.P., Chadha G.S., Klotz D., Visvanathan A., Lamond A., Swedlow J.R., Nathke I.S. (2012). The adenomatous polyposis coli protein contributes to normal compaction of mitotic chromatin. PloS One.

[bib30] du Pasquier L., Robert J. (1992). *In vitro* growth of thymic tumor cell lines from *Xenopus*. Dev. Immunol..

[bib31] Dworkin M.B., Dworkin-Rastl E. (1989). Metabolic regulation during early frog development: glycogenic flux in *Xenopus* oocytes, eggs, and embryos. Dev. Biol..

[bib32] Earley E.M., Reinschmidt D.C., Tompkins R., Gebhardt B.M. (1995). Tissue culture of a mixed cell thymic tumor from *Xenopus laevis. In vitro* cellular and developmental biology. Animal.

[bib33] Felix M.A., Labbe J.C., Doree M., Hunt T., Karsenti E. (1990). Triggering of cyclin degradation in interphase extracts of amphibian eggs by cdc2 kinase. Nature.

[bib34] Felix M.A., Pines J., Hunt T., Karsenti E. (1989). A post-ribosomal supernatant from activated *Xenopus* eggs that displays post-translationally regulated oscillation of its cdc2+ mitotic kinase activity. EMBO J.

[bib35] Glackin C.A. (2014). Targeting the Twist and Wnt signaling pathways in metastatic breast cancer. Maturitas.

[bib36] Goding C.R., Pei D., Lu X. (2014). Cancer: pathological nuclear reprogramming? Nature reviews. Cancer.

[bib37] Goyos A., Robert J. (2009). Tumorigenesis and anti-tumor immune responses in *Xenopus*. Front. Biosci. (Landmark edition).

[bib38] Gradl D., Kuhl M., Wedlich D. (1999). Keeping a close eye on Wnt-1/wg signaling in *Xenopus*. Mech. Dev..

[bib39] Gurdon J.B., Elsdale T.R., Fischberg M. (1958). Sexually mature individuals of *Xenopus laevis* from the transplantation of single somatic nuclei. Nature.

[bib40] Halley-Stott R.P., Gurdon J.B. (2013). Epigenetic memory in the context of nuclear reprogramming and cancer. Brief. Funct. Genomics.

[bib41] Halley-Stott R.P., Pasque V., Gurdon J.B. (2013). Nuclear reprogramming. Development.

[bib42] Hanahan D., Weinberg R.A. (2000). The hallmarks of cancer. Cell.

[bib43] Hanahan D., Weinberg R.A. (2011). Hallmarks of cancer: the next generation. Cell.

[bib44] Hardwick L.J., Philpott A. (2014). Nervous decision-making: to divide or differentiate. Trends Genet..

[bib45] Harper J.V., Brooks G. (2005). The mammalian cell cycle: an overview. Methods Mol. Biol..

[bib46] Hashimoto Y., Costanzo V. (2011). Studying DNA replication fork stability in *Xenopus* egg extract. Methods Mol. Biol..

[bib47] Haynes-Gilmore N., Banach M., Edholm E.S., Lord E., Robert J. (2014). A critical role of non-classical MHC in tumor immune evasion in the amphibian *Xenopus* model. Carcinogenesis.

[bib48] Hikasa H., Sokol S.Y. (2013). Wnt signaling in vertebrate axis specification. Cold Spring Harb. Perspect. Biol..

[bib49] Hindley C., Ali F., McDowell G., Cheng K., Jones A., Guillemot F., Philpott A. (2012). Post-translational modification of Ngn2 differentially affects transcription of distinct targets to regulate the balance between progenitor maintenance and differentiation. Development.

[bib50] Hingorani S.R., Wang L., Multani A.S., Combs C., Deramaudt T.B., Hruban R.H., Rustgi A.K., Chang S., Tuveson D.A. (2005). Trp53R172H and KrasG12D cooperate to promote chromosomal instability and widely metastatic pancreatic ductal adenocarcinoma in mice. Cancer Cell.

[bib51] Ishimura A., Lee H.S., Bong Y.S., Saucier C., Mood K., Park E.K., Daar I.O. (2006). Oncogenic Met receptor induces ectopic structures in *Xenopus* embryos. Oncogene.

[bib52] Joukov V., Groen A.C., Prokhorova T., Gerson R., White E., Rodriguez A., Walter J.C., Livingston D.M. (2006). The BRCA1/BARD1 heterodimer modulates ran-dependent mitotic spindle assembly. Cell.

[bib53] Kalluri R., Weinberg R.A. (2009). The basics of epithelial–mesenchymal transition. J. Clin. Investig..

[bib54] Kuhl M. (2002). Non-canonical Wnt signaling in *Xenopus*: regulation of axis formation and gastrulation. Semin. Cell Dev. Biol..

[bib55] Kuhl M., Pandur P. (2008). Dorsal axis duplication as a functional readout for Wnt activity. Methods Mol. Biol..

[bib56] Kuhl M., Pandur P. (2008). Measuring CamKII activity in *Xenopus* embryos as a read-out for non-canonical Wnt signaling. Methods Mol. Biol..

[bib57] Kvist T., Hansen K.B., Brauner-Osborne H. (2011). The use of *Xenopus* oocytes in drug screening. Expert Opin. Drug Discov..

[bib58] Laiho M., Latonen L. (2003). Cell cycle control, DNA damage checkpoints and cancer. Ann. Med..

[bib59] Landais I., Sobeck A., Stone S., la Chapelle A., Hoatlin M.E. (2009). A novel cell-free screen identifies a potent inhibitor of the *Fanconi anemia* pathway.. Int. J. Cancer/J. Int. Cancer.

[bib60] Lander R., Nasr T., Ochoa S.D., Nordin K., Prasad M.S., Labonne C. (2013). Interactions between Twist and other core epithelial–mesenchymal transition factors are controlled by GSK3-mediated phosphorylation. Nat. Commun..

[bib61] Laskey R.A., Honda B.M., Mills A.D., Morris N.R., Wyllie A.H., Mertz J.E., De Roberts E.M., Gurdon J.B. (1978). Chromatin assembly and transcription in eggs and oocytes of *Xenopus laevis*. Cold Spring Harb. Symp. Quant. Biol..

[bib62] Leno G.H., Downes C.S., Laskey R.A. (1992). The nuclear membrane prevents replication of human G2 nuclei but not G1 nuclei in *Xenopus* egg extract. Cell.

[bib63] Leno G.H., Laskey R.A. (1991). DNA replication in cell-free extracts from *Xenopus laevis*. Methods Cell Biol..

[bib64] Liu D., Rudland P.S., Sibson D.R., Platt-Higgins A., Barraclough R. (2005). Human homologue of cement gland protein, a novel metastasis inducer associated with breast carcinomas. Cancer Res..

[bib65] Liu X., Chang Y., Reinhart P.H., Sontheimer H., Chang Y. (2002). Cloning and characterization of glioma BK, a novel BK channel isoform highly expressed in human glioma cells. J. Neurosci. Off. J. Soc. Neurosci..

[bib66] Lujan E., Wernig M. (2012). The many roads to Rome: induction of neural precursor cells from fibroblasts. Curr. Opin. Genet. Dev..

[bib67] Ma Y., Zhang P., Wang F., Yang J., Yang Z., Qin H. (2010). The relationship between early embryo development and tumourigenesis. J. Cell. Mol. Med..

[bib68] Mani S.A., Yang J., Brooks M., Schwaninger G., Zhou A., Miura N., Kutok J.L., Hartwell K., Richardson A.L., Weinberg R.A. (2007). Mesenchyme Forkhead 1 (FOXC2) plays a key role in metastasis and is associated with aggressive basal-like breast cancers. Proc. Natl. Acad. Sci. USA.

[bib69] McDowell G.S., Hardwick L.J., Philpott A. (2014). Complex domain interactions regulate stability and activity of closely related proneural transcription factors. Biochem. Biophys. Res. Commun..

[bib70] Mimori-Kiyosue Y., Matsui C., Sasaki H., Tsukita S. (2007). Adenomatous polyposis coli (APC) protein regulates epithelial cell migration and morphogenesis via PDZ domain-based interactions with plasma membranes. Genes Cells: Devot. Mol Cell Mech.

[bib71] Minshull J., Golsteyn R., Hill C.S., Hunt T. (1990). The A- and B-type cyclin associated cdc2 kinases in *Xenopus* turn on and off at different times in the cell cycle. EMBO J..

[bib72] Miyata S., Wang L.Y., Wang N.L., Yao X.S., Kitanaka S. (2004). Selective inhibition of the growth of cancer cells by diterpenes selected with embryonic cells of *Xenopus*. Cell Biol. Int..

[bib73] Nakaya Y., Sheng G. (2013). EMT in developmental morphogenesis. Cancer Lett..

[bib74] Newport J., Kirschner M. (1982). A major developmental transition in early *Xenopus* embryos: I. characterization and timing of cellular changes at the midblastula stage. Cell.

[bib75] Newport J., Kirschner M. (1982). A major developmental transition in early *Xenopus* embryos: II. Control of the onset of transcription. Cell.

[bib76] Nutt L.K. (2012). The *Xenopus* oocyte: a model for studying the metabolic regulation of cancer cell death. Semin. Cell Dev. Biol..

[bib77] Ny A., Koch M., Schneider M., Neven E., Tong R.T., Maity S., Fischer C., Plaisance S., Lambrechts D., Heligon C., Terclavers S., Ciesiolka M., Kalin R., Man W.Y., Senn I., Wyns S., Lupu F., Brandli A., Vleminckx K., Collen D., Dewerchin M., Conway E.M., Moons L., Jain R.K., Carmeliet P. (2005). A genetic *Xenopus laevis* tadpole model to study lymphangiogenesis. Nat. Med..

[bib78] Ny A., Vandevelde W., Hohensinner P., Beerens M., Geudens I., Diez-Juan A., Brepoels K., Plaisance S., Krieg P.A., Langenberg T., Vinckier S., Luttun A., Carmeliet P., Dewerchin M. (2013). A transgenic *Xenopus laevis* reporter model to study lymphangiogenesis. Biol. Open.

[bib79] Okudaira H., Nakanishi T., Oka S., Kobayashi M., Tamagami H., Schuster D.M., Goodman M.M., Shirakami Y., Tamai I., Kawai K. (2013). Kinetic analyses of trans-1-amino-3-[18F]fluorocyclobutanecarboxylic acid transport in *Xenopus laevis* oocytes expressing human ASCT2 and SNAT2. Nucl. Med. Biol..

[bib80] Ortega S., Prieto I., Odajima J., Martin A., Dubus P., Sotillo R., Barbero J.L., Malumbres M., Barbacid M. (2003). Cyclin-dependent kinase 2 is essential for meiosis but not for mitotic cell division in mice. Nat. Genet..

[bib81] Pakladok T., Hosseinzadeh Z., Alesutan I., Lang F. (2012). Stimulation of the Na(+)-coupled glucose transporter SGLT1 by B-RAF. Biochem. Biophys. Res. Commun..

[bib82] Pakladok T., Hosseinzadeh Z., Almilaji A., Lebedeva A., Shumilina E., Alesutan I., Lang F. (2014). Up-regulation of hERG K(+) channels by B-RAF. PloS One.

[bib83] Pennisi E. (1998). How a growth control path takes a wrong turn to cancer. Science.

[bib84] Philpott A., Yew P.R. (2008). The *Xenopus* cell cycle: an overview. Mol. Biotechnol..

[bib85] Rane S.G., Dubus P., Mettus R.V., Galbreath E.J., Boden G., Reddy E.P., Barbacid M. (1999). Loss of Cdk4 expression causes insulin-deficient diabetes and Cdk4 activation results in beta-islet cell hyperplasia. Nat. Genet..

[bib86] Richard-Parpaillon L., Cosgrove R.A., Devine C., Vernon A.E., Philpott A. (2004). G1/S phase cyclin-dependent kinase overexpression perturbs early development and delays tissue-specific differentiation in *Xenopus*. Development.

[bib87] Robert J. (2010). Comparative study of tumorigenesis and tumor immunity in invertebrates and nonmammalian vertebrates. Dev. Comp. Immunol..

[bib88] Robert J., Cohen N. (1998). Evolution of immune surveillance and tumor immunity: studies in *Xenopus*. Immunol. Rev..

[bib89] Robert J., Guiet C., Cohen N., du Pasquier L. (1997). Effects of thymectomy and tolerance induction on tumor immunity in adult *Xenopus laevis*.. Int. J. Cancer/J. Int. Cancer.

[bib90] Robert J., Guiet C., Du Pasquier L. (1994). Lymphoid tumors of *Xenopus laevis* with different capacities for growth in larvae and adults. Dev. Immunol..

[bib91] Rosania G.R., Merlie J., Gray N., Chang Y.T., Schultz P.G., Heald R. (1999). A cyclin-dependent kinase inhibitor inducing cancer cell differentiation: biochemical identification using *Xenopus* egg extracts. Proc. Natl. Acad. Sci.USA.

[bib92] Ruben L.N., Clothier R.H., Balls M. (2007). Cancer resistance in amphibians. Altern. Lab. Anim..

[bib93] Sadikovic B., Al-Romaih K., Squire J.A., Zielenska M. (2008). Cause and consequences of genetic and epigenetic alterations in human cancer. Curr. Genomics.

[bib94] Saka Y., Smith J.C. (2001). Spatial and temporal patterns of cell division during early *Xenopus* embryogenesis. Dev. Biol..

[bib95] Santamaria D., Ortega S. (2006). Cyclins and CDKS in development and cancer: lessons from genetically modified mice. Front. Biosci. J. Virtual Libr..

[bib96] Schmitt S.M., Gull M., Brandli A.W. (2014). Engineering *Xenopus* embryos for phenotypic drug discovery screening. Adv. Drug Deliv. Rev..

[bib97] Srinivasan S.V., Gautier J. (2011). Study of cell cycle checkpoints using *Xenopus* cell-free extracts. Methods Mol. Biol..

[bib98] Taube J.H., Herschkowitz J.I., Komurov K., Zhou A.Y., Gupta S., Yang J., Hartwell K., Onder T.T., Gupta P.B., Evans K.W., Hollier B.G., Ram P.T., Lander E.S., Rosen J.M., Weinberg R.A., Mani S.A. (2010). Core epithelial-to-mesenchymal transition interactome gene-expression signature is associated with claudin-low and metaplastic breast cancer subtypes. Proc. Natl. Acad. Sci. USA.

[bib99] Theveneau E., Mayor R. (2012). Neural crest delamination and migration: from epithelium-to-mesenchyme transition to collective cell migration. Dev. Biol..

[bib100] Thiery J.P., Acloque H., Huang R.Y., Nieto M.A. (2009). Epithelial–mesenchymal transitions in development and disease. Cell.

[bib101] Tsutsui T., Hesabi B., Moons D.S., Pandolfi P.P., Hansel K.S., Koff A., Kiyokawa H. (1999). Targeted disruption of CDK4 delays cell cycle entry with enhanced p27(Kip1) activity. Mol. Cell. Biol..

[bib102] Tumova L., Pombinho A.R., Vojtechova M., Stancikova J., Gradl D., Krausova M., Sloncova E., Horazna M., Kriz V., Machonova O., Jindrich J., Zdrahal Z., Bartunek P., Korinek V. (2014). Monensin inhibits canonical Wnt signaling in human colorectal cancer cells and suppresses tumor growth in multiple intestinal neoplasia mice. Mol. Cancer Ther..

[bib103] Ureta T., Preller A., Kessi E. (2001). Frog oocytes: a living test tube for studies on metabolic regulation. IUBMB Life.

[bib104] Vernon A.E. (2003). The cdk inhibitor p27Xic1 is required for differentiation of primary neurones in *Xenopus*. Development.

[bib105] Vernon A.E., Devine C., Philpott A. (2003). The cdk inhibitor p27Xic1 is required for differentiation of primary neurones in *Xenopus*. Development.

[bib106] Vernon A.E., la Bonne C. (2004). Tumor metastasis: a new twist on epithelial–mesenchymal transitions. Curr. Biol..

[bib107] Vernon A.E., Movassagh M., Horan I., Wise H., Ohnuma S., Philpott A. (2006). Notch targets the Cdk inhibitor Xic1 to regulate differentiation but not the cell cycle in neurons. EMBO Rep..

[bib108] Vernon A.E., Philpott A. (2003). A single cdk inhibitor, p27Xic1, functions beyond cell cycle regulation to promote muscle differentiation in Xenopus. Development.

[bib109] Vosper J.M., McDowell G.S., Hindley C.J., Fiore-Heriche C.S., Kucerova R., Horan I., Philpott A. (2009). Ubiquitylation on canonical and non-canonical sites targets the transcription factor neurogenin for ubiquitin-mediated proteolysis. J. Biol. Chem..

[bib110] Waaler J., Machon O., Tumova L., Dinh H., Korinek V., Wilson S.R., Paulsen J.E., Pedersen N.M., Eide T.J., Machonova O., Gradl D., Voronkov A., von Kries J.P., Krauss S. (2012). A novel tankyrase inhibitor decreases canonical Wnt signaling in colon carcinoma cells and reduces tumor growth in conditional APC mutant mice. Cancer Res..

[bib111] Waaler J., Machon O., von Kries J.P., Wilson S.R., Lundenes E., Wedlich D., Gradl D., Paulsen J.E., Machonova O., Dembinski J.L., Dinh H., Krauss S. (2011). Novel synthetic antagonists of canonical Wnt signaling inhibit colorectal cancer cell growth. Cancer Res..

[bib112] Wallingford J.B. (1999). Tumors in tadpoles: the *Xenopus* embryo as a model system for the study of tumorigenesis. Trends Genet: TIG.

[bib113] Wallingford J.B., Seufert D.W., Virta V.C., Vize P.D. (1997). p53 activity is essential for normal development in *Xenopus*. Curr. Biol..

[bib114] Wang Y. (2009). Wnt/Planar cell polarity signaling: a new paradigm for cancer therapy. Mol. Cancer Ther..

[bib115] Wang Z., Gao H., Wang H., Ren X., Bao L., Sa R., Wang J., Bai H., Yu H. (2013). Specific reversal of tumor-suppressor gene promoter hypermethylation with bovine oocyte extract. Oncol. Rep..

[bib116] Wang Z., Yue Y., Han P., Sa R., Ren X., Wang J., Bai H., Yu H. (2013). Remodeling epigenetic modifications at tumor suppressor gene promoters with bovine oocyte extract. Cytotherapy.

[bib117] Wei X., Sun H., Yan H., Zhang C., Zhang S., Liu X., Hua N., Ma X., Zheng J. (2013). ZC88, a novel 4-amino piperidine analog, inhibits the growth of neuroblastoma cells through blocking hERG potassium channel. Cancer Biol. Ther..

[bib118] Whitman M., Melton D.A. (1992). Involvement of p21ras in *Xenopus* mesoderm induction. Nature.

[bib119] Willis J., de Stephanis D., Patel Y., Gowda V., Yan S. (2012). Study of the DNA damage checkpoint using *Xenopus* egg extracts. J. Vis. Exp..

[bib120] Woodland H.R. (1974). Some studies on early embryonic development relevant to the study of cancer. J. Clin. Pathol..

[bib121] Wu F., Stutzman A., Mo Y.Y. (2007). Notch signaling and its role in breast cancer. Front. Biosci. J. Virtual Libr..

[bib122] Wu X., Gao H., Ke W., Hager M., Xiao S., Freeman M.R., Zhu Z. (2011). VentX trans-activates p53 and p16ink4a to regulate cellular senescence. J. Biol. Chem..

[bib123] Xi Y., Chen Y. (2014). Wnt signaling pathway: implications for therapy in lung cancer and bone metastasis. Cancer Lett..

[bib124] Xie K., Abbruzzese J.L. (2003). Developmental biology informs cancer: the emerging role of the hedgehog signaling pathway in upper gastrointestinal cancers. Cancer Cell.

[bib125] Yang J., Mani S.A., Donaher J.L., Ramaswamy S., Itzykson R.A., Come C., Savagner P., Gitelman I., Richardson A., Weinberg R.A. (2004). Twist, a master regulator of morphogenesis, plays an essential role in tumor metastasis. Cell.

[bib126] Yang S., Lockwood A., Hollett P., Ford R., Kao K. (1998). Overexpression of a novel *Xenopus* Rel mRNA Gene induces tumors in early embryos. J. Biol. Chem..

[bib127] Yew P.R., Kirschner M.W. (1997). Proteolysis and DNA replication: the CDC34 requirement in the *Xenopus* egg cell cycle. Science.

[bib128] You Z., Bailis J.M., Johnson S.A., Dilworth S.M., Hunter T. (2007). Rapid activation of ATM on DNA flanking double-strand breaks. Nat. Cell Biol..

[bib129] Zylkiewicz E., Stukenberg P.T. (2014). *Xenopus* egg extracts as a simplified model system for structure–function studies of dynein regulators. Methods Mol. Biol..

